# Voluntary Aid in War

**Published:** 1909-08-21

**Authors:** 


### Voluntary Aid in War.

The Army Council, as announced in the daily papers on August 17, has had under consideration the provision of organisation for the relief of the sick and wounded during home defence; and has drawn up and issued a scheme for such voluntary aid in England and Wales, which has been issued to Territorial County Associations throughout the country. By this scheme the County Associations are charged with the responsibility of the organisation of voluntary aid in each county, and are recommended through the local branches of the British Red Cross Society to form "voluntary aid detachments" with an establishment which is laid down in the scheme: each detachment to be capable of being used as a clearing hospital, a rest station, or as the personnel of an ambulance train, as the circumstances of the moment may demand in time of war. The training and equipment of these voluntary aid detachments is to be carried out in whatever way the British Red Cross Society thinks fit; but they should be so arranged as to be able to make use of local resources for improvising accommodation for and transport of wounded to the general hospitals. They are, in fact, to fill the existing gap, at present quite unbridged, between the field ambulances and the general hospitals. Since the former are intended to be essentially mobile units, capable of transporting themselves at a moment's notice to the scene of action, it is evident that the solution of the problem of the evacuation of their patients rapidly and successfully is vital to the whole organisation of medical aid in time of war. To utilise the Red Cross Society to fill this gap seems to us a mistake. Dictated probably by the financial starvation which is threatening the very life of the Territorial scheme, this policy of wedging in an amateur, non-disciplined, and untrained link between the field units and the general hospitals seems to be much of a piece with the strictly paper equipment of the two ends of the chain, and just as likely to break down seriously when the strain comes. It is not fair or desirable that a voluntary society such as the Red Cross should be expected to equip, as well as to raise the personnel of, such essential parts of the organisation of our medical service for war as the "clearing" and "stationary" hospitals. This is the duty of the War Office, and to no outside corporation should it be delegated. It is some satisfaction to see it authoritatively recognised that something must be done, but much less so to note the half-hearted and niggardly way in which the provision of the remedy is set about. The Red Cross Society, and the St. John's and St. Andrew's Ambulance Societies, too, might well be entrusted with the care of those who are well enough to be discharged from the stationary and general hospitals but unfit immediately to resume their places in the firing line. But the proper place for a clearing hospital is within a few hours' march of the firing-line, and even on occasions almost within gunshot of it; and we must repeat that the chaos of the modern battlefield, and especially the horrors of its hospitals, are more likely to be accentuated than diminished by the intrusion of untrained and unequipped amateurs of both sexes into the midst of operations.

